# Biofilm Formation, Pyocyanin Production, and Antibiotic Resistance Profile of *Pseudomonas aeruginosa* Isolates from Wounds

**DOI:** 10.1155/2024/1207536

**Published:** 2024-02-20

**Authors:** Larissa Yetendje Chimi, Michel Noubom, Borel Ndezo Bisso, Guy Sedar Singor Njateng, Jean Paul Dzoyem

**Affiliations:** ^1^Department of Biochemistry, Faculty of Science, University of Dschang, Dschang, Cameroon; ^2^Department of Microbiology, Hematology and Immunology, Faculty of Medicine and Pharmaceutical Sciences, University of Dschang, Dschang, Cameroon

## Abstract

*Pseudomonas aeruginosa* is one of the most frequently resistant and dangerous bacteria isolated from infected wounds of patients. This study aimed to determine the prevalence of *P. aeruginosa* from infected wounds of patients in the Dschang District Hospital to evaluate their antibiotic susceptibility profiles and their ability to swarm and swim and correlate pyocyanin production with biofilm formation. Wound swab samples were collected and the identification of *P. aeruginosa* was performed using microbiological and biochemical tests. Their antimicrobial susceptibility was determined by the broth microdilution method. Swarming and swimming were determined by measuring the diameters of motility in semisolid/low-viscosity media. Furthermore, pyocyanin production and biofilm formation were evaluated spectrophotometrically using a microtiter plate. The prevalence of *P. aeruginosa* from infected wounds in our study population was 26%. All *P. aeruginosa* isolates were resistant to streptomycin and paromomycin, and the frequency of multidrug resistance (MDR) was 65.8%. All *P. aeruginosa* isolates showed the ability to produce biofilm and pyocyanin. Out of the 37 isolates screened, 19 including the reference strains (51.4%) were strong biofilm producers. A significant positive correlation was observed among biofilm formation, pyocyanin production, and the antibiotic resistance profile of the isolates. Findings from this study suggest that infected wounds could act as a reservoir for MDR and virulent *P. aeruginosa*. The presence of strong biofilm producers of *P. aeruginosa* in infected wounds is a serious public health concern. Therefore, surveillance programs to monitor and control MDR *P. aeruginosa* in these patients are required to prevent their dissemination in hospital settings.

## 1. Introduction

*P. aeruginosa* is the most important Gram-negative opportunistic pathogen and a major cause of nosocomial infection [1]. It is a causative agent for 10% of all hospital-acquired infections, including pulmonary infections (tracheobronchitis and necrotizing bronchopneumonia), urinary tract infections, bacteremia, endocarditis, and skin and soft-tissue infections such as surgical and burns wounds site infections [2, 3].

Wounds provide a suitable site for bacterial multiplication and are persistent sources of infection [4]. In infected wounds, *P. aeruginosa* was found to be the most common isolate (59%) [5]. Other bacteria involved in the development of chronicity and delaying wound healing included species such as *Staphylococcus aureus*, *Proteus mirabilis*, *Escherichia coli*, and *Klebsiella pneumoniae* [6]. In 2017, *P. aeruginosa* was recognized as one of the most life-threatening bacteria and listed as a priority pathogen for research and development of new antibiotics by the World Health Organization [7]. A previous study showed that 61–100% of *P. aeruginosa* observed in intensive care units in the majority of countries in the Middle East and North Africa region are multiresistant [8]. *P. aeruginosa* possesses numerous virulence factors and has the potential to develop resistance to antimicrobials [2]. The bacterium produces a variety of virulence factors, such as *Pseudomonas* quinolone signal (PQS), pyocyanin, rhamnolipids, elastase, and two endogenous siderophores, pyoverdine and pyochelin, which are involved in chronic infections [9]. Pyocyanin, a blue-green pigment characteristic of *P. aeruginosa* is an electrochemically active metabolite, involved in various biological activities such as biofilm formation, maintaining the fitness of bacterial cells, and gene expression [10]. It is a redox-active blue phenazine pigment and the redox equilibrium inside a biological system is altered by pyocyanin [11]. Pyocyanin has long been suspected to play a role in the pathogenesis. The production of this pigment enhances the expression of virulence factors and other phenotypes that converge on *P. aeruginosa*. It has been reported that *P. aeruginosa* strains that exhibit pyocyanin have a prevalence of higher multidrug resistance, as well as more virulence factors, compared to nonproducing strains [12].

Bacterial motility plays an important role in surface colonization by bacteria [13]. In addition to flagellum-dependent swimming in liquid-to-low viscosity environments, *P. aeruginosa* is capable of swarming motility, which occurs on semisolid surfaces and requires flagellar motility as well as the production of biosurfactants [14]. These motilities contribute to the formation of structured surface-associated communities of bacteria called biofilms.

Biofilm is defined as an assemblage of microbial cells that are attached to a surface and enclosed in an extracellular matrix principally made of polysaccharide material [15–17]. Biofilm is considered an important virulence factor and plays a significant role in antibiotic resistance [18]. Biofilm formation is a strategy of microorganisms to successfully adapt and survive in hostile environments which can increase their resistance to the effects of antimicrobial agents 10–1000 times [19–21]. The prevalence of biofilms in chronic wounds is estimated to be approximately 60% [22]. Furthermore, biofilms account for almost 80% of chronic microbial infections in the human body. Previous works have shown the involvement of pyocyanin and biofilm formation in the bacterial resistance of *P. aeruginosa* isolates from other clinical samples [23]. To date, little or no information is known on the distribution of *P. aeruginosa* isolates from infected wounds, as well as the resistance phenotype of the isolates, production of pyocyanin, and biofilm formation in Cameroon, especially in the West Region of Cameroon. In this light, this study aimed to determine the prevalence of *P. aeruginosa* from infected wounds of patients at the Dschang District Hospital, evaluating their antibiotic resistance profile. Furthermore, we evaluated the pathogenicity of *P. aeruginosa* by assessing biofilm formation and pyocyanin production, as well as the swarming and swimming ability of the isolates.

## 2. Materials and Methods

### 2.1. Population Study and Sample Collection

#### 2.1.1. Study Design and Ethical Considerations

A cross-sectional study was conducted on 142 patients with infected wounds. This was done by visiting the outpatient units of the Department of Wound Care for a period of 3 months from February 2022 to April 2022 at the Dschang District Hospital, Cameroon. Any patient who was immunocompromised, under treatment with antibiotics, or who had not given his consent was not included in this study.

This study was approved by the Institutional Ethics Committee for Research on Human Health with reference number 2961 CEI-Udo/02/2021/T. The samples were collected following international safety rules and ethical standards. Informed and written consent and assent were obtained from the study participants before data collection.

#### 2.1.2. Sample Collection, Isolation, and Identification of Isolates

A total of 142 clinical samples were collected from the infected wounds of patients under aseptic conditions with the help of sterile cotton swabs. For patients with dry wounds, the swab was moistened with a sterile saline solution before swabbing. The samples collected were then transferred to the research unit of Microbiology and Antimicrobial Substances at the Department of Biochemistry of the University of Dschang for analysis. The samples collected on the swabs were then cultured on Mueller–Hinton agar at 37°C for 24 h for bacterial growth and isolation. Identification of the *Pseudomonas aeruginosa* isolate was performed by standard microbiological and biochemical methods, including inoculation on a cetrimide agar medium. *Pseudomonas aeruginosa* isolates were characterized based on colony morphology, pigmentation on the King A agar medium, and the ability of the bacterium to produce oxidase, catalase, citrate, and urea-indole.

### 2.2. Antibiotic Susceptibility Test

The antibiotics used for susceptibility testing belonged to the following four classes: a 3^rd^ cephalosporin (ceftazidime), four aminoglycosides (amikacin, paromomycin, streptomycin, and neomycin), and a quinolone (norfloxacin). All were purchased from Sigma-Aldrich, China. *In vitro* antibacterial susceptibility testing was performed by the broth dilution method with Mueller–Hinton broth (Merck, Germany) according to the European Committee on Antimicrobial Susceptibility Testing (EUCAST) guidelines [24]. *P. aeruginosa* ATCC 27853 was used as the reference strain. The multidrug-resistant *P. aeruginosa* (MDR-Pa) was defined as an isolate resisting more than one antimicrobial agent in three or more antimicrobial categories or classes [25].

### 2.3. Biofilm Formation Assay

Biofilm formation assay was performed by the microtiter plate method described by Kirmusaoğlu and Kaşikçi [26] with some modifications. In brief, 100 *μ*L of Mueller–Hinton broth (MHB) supplemented with 1% glucose and 100 *μ*L of inoculum (1.5 × 10^6^ CFU/mL) were inoculated into 96-well flat-bottomed sterile polystyrene microplates. Microplates were then incubated at 37°C for 24 h, 48 h, and 72 h. After each time of incubation, planktonic cells in the well of the microplate were removed. Each well was washed three times with 200 *μ*L of sterile phosphate-buffered saline (pH 7.2). After washing, 150 *μ*L of methanol was introduced into each well and allowed for 20 min to fix the bacteria. The microtiter plates were then emptied by simple flicking. The biofilm was stained for 15 min using 150 *μ*L of safranin 1%. Subsequently, the cell-bound dye was resolubilized with 150 *μ*L of 95% ethanol per well. The microtiter plates were covered (to minimize evaporation) and left at room temperature for 30 min. Then, the optical density (OD) was measured at 570 nm by a Versamax tunable microplate reader. The study was performed in quadruplets and repeated three times. The uninoculated wells containing sterile MHB supplemented with 1% glucose were considered as negative controls and used as blanks. Wells in which isolates had an OD greater than that of the blank were considered as biofilm producers. We then calculated the cutoff values by using the following formula:

(1) $$\begin{array}{c} \text{OD control}\left(\text{ODc}\right)=\text{mean OD of negative control}+\left(3\times \text{standard deviation}\left(\text{SD}\right)\text{of negative control}\right),\\ OD\text{ isolate}=\text{mean OD of isolate }-\text{ODc}. \end{array}$$

The results were interpreted into four categories as follows: OD ≤ ODc: no biofilm production (NBP); ODc < OD ≤ 2 × ODc: weak biofilm production (WBP); 2 × ODc < OD ≤ 4 × ODc: moderate biofilm production (MBP); and 4 × ODc < OD: strong biofilm production (SBP) [26].

### 2.4. Pyocyanin Quantification Assay

Pyocyanin production by *P. aeruginosa* was determined by the method described by Alayande et al. with slight modifications [27]. In brief, the isolates were incubated in 10 mL of Luria–Bertani medium at 37°C for 24 h, 48 h, and 72 h. After each time of incubation, the cultured cells were centrifuged at 1500 × *g* for 10 min to separate the supernatants from the pellet after which 3 mL of chloroform were added to 5 mL of the supernatant and the mixture was agitated vigorously using a vortex mixer. At the chloroform layer, 1 mL of 0.2 M HCl was added and then centrifuged at 1500 × *g* for 10 min. The optical density of the HCl layer was measured at 520 nm using a spectrophotometer (Biobase BK-D590 Double Beam Scanning UV/Vis; China). The HCl 0.2 M was used as a control (Blank). The concentration of pyocyanin was obtained using the following formula: concentration of pyocyanin (µg/mL)=(DO_520nm_  − DO_blank_)*∗*17.072.

### 2.5. Swarming and Swimming Motility Assays

The swarming motility test was determined following the method described by O'May and Tufenkji with a few modifications [13]. In brief, LB broth with 1% agar sterilized in the autoclave at 121°C for 15 min was poured into 82 mm noncompartmentalized Petri dishes. A total of 2.5 *μ*L of bacterial suspension in broth standardized at 0.5 McFarland aged 18–24 h previously cultured in the LB medium were gently deposited on the surface of the agar (at the center), allowing the visualization of the motility zone on the agar surface. Dishes were sealed and placed in incubators for 24 h, 48 h, and 72 h. At the end of each incubation period, the diameter of the motility zone was measured.

The swimming motility test was determined according to the method described above but with some variations. The previously sterilized LB broth with 0.5% agar was poured into the 82 mm Petri dishes. The bacterial broth was deposited at the center of the agar (and not at the surface). Dishes were sealed and placed in incubators for 24 h, 48 h, and 72 h. At the end of each incubation period, the diameter of the motility zone was measured.

### 2.6. Statistical Analysis

The Pearson correlation test was used to evaluate the relationship between pyocyanin production and biofilm formation with antimicrobial susceptibility. Data analyses were performed using GraphPad Prism 8.0. *P* < 0.05 was considered statistically significant.

## 3. Results

### 3.1. Prevalence of *P. aeruginosa* in Infected Wounds

A total of 37 isolates of *P. aeruginosa* were identified from 142 clinical samples collected from infected wounds at the district hospital of Dschang, resulting in a prevalence of 26.1%.

### 3.2. Antibiotic Susceptibility Test

**Figure 1:**
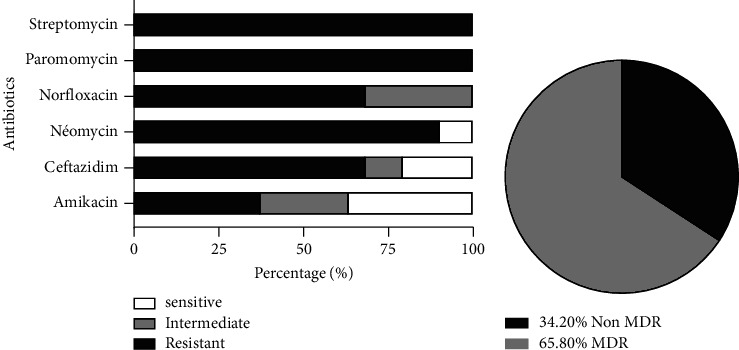
Antibiotic susceptibility (a) and resistance phenotype (b) patterns of *P. aeruginosa* isolates recovered from wounds.

The susceptibility of *P. aeruginosa* isolates to different antibiotics tested and the different phenotypes of isolates obtained are shown in Figure 1. In our study, 38 isolates of *P. aeruginosa* showed 100% resistance to streptomycin and paromomycin, 14 (36.8%) isolates showed resistance to amikacin, and 26 (68.4%) isolates showed resistance to ceftazidime. The different phenotypes of the isolates are represented in Figure 1(b). Out of the 38 isolates of *P. aeruginosa*, 25 isolates showed multidrug resistance with a prevalence of 65.8%.

### 3.3. Biofilm Formation Ability of *P. aeruginosa* Isolates

**Figure 2:**
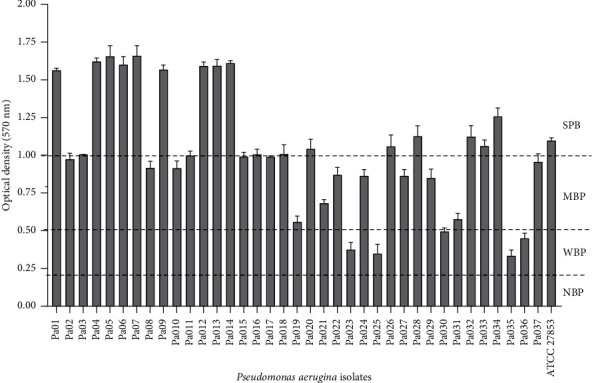
Biofilm forming ability of *P. aeruginosa* isolates recovered from wounds (NBP: nonbiofilm producers, WBP: weak biofilm producers, MBP: moderate biofilm producers, and SBP: strong biofilm producers).

The ability of different isolates of *P. aeruginosa* to form biofilms was evaluated at different times (24 h, 48 h, and 72 h). *P. aeruginosa* showed the ability to form biofilms, which was successfully quantified by the safranin dye (Supplementary material S1). The biofilm formation capacity increased with the incubation time up to 48 h and a decrease was observed after 72 h (Supplementary material S2). The best time of biofilm formation in all isolates of *P. aeruginosa* isolates was observed at 48 h (Figure 2). Out of the 38 isolates, 50% (19 isolates) and 37% (14 isolates) were strong and moderate biofilm producers, respectively, with optical densities ranging from 1 to 1.7 and 0.5 to 1. Finally, 13% of the isolates (5 isolates) were classified as weak biofilm producers.

### 3.4. Pyocyanin Production by *P. aeruginosa* Isolates

**Figure 3:**
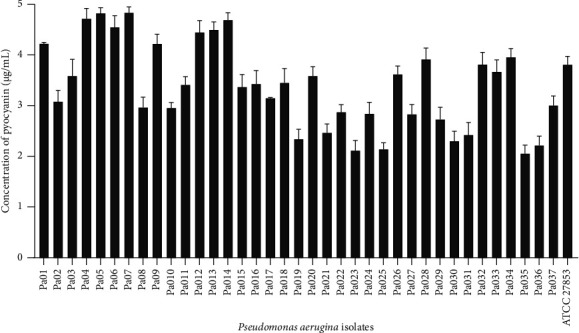
Pyocyanin production by *P. aeruginosa* isolates recovered from wounds.

The capacity of isolates to produce pyocyanin is shown in Figure 3. Pyocyanin production was actively higher at 72 h of incubation (Supplementary material S3). All isolates of *P. aeruginosa* isolates were producers of pyocyanin with concentrations ranging from 2.1 to 4.4 *μ*g/mL.

### 3.5. Swarming and Swimming Motility Ability of *P. aeruginosa* Isolates

**Figure 4:**
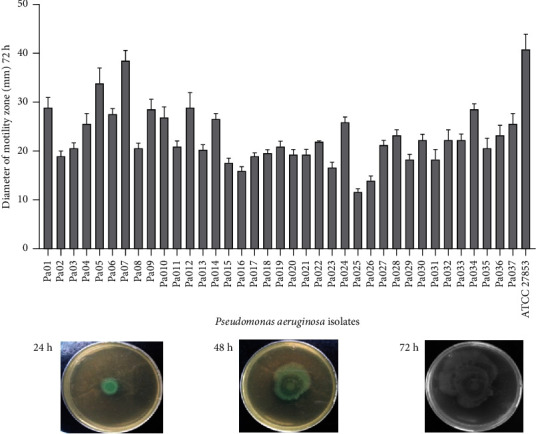
Swarming motility of *P. aeruginosa*. 2.5 *μ*L of bacterial suspension was dropped at the center surface of the agar plate and incubated for 24 h, 48 h, and 72 h at 37°C. In this figure are photographs of representative swarming motility at different time intervals. The diameter of the swarming motility zone increased with incubation time.

The swarming activity of the isolates is shown in Figure 4. All isolates showed the ability to move in semisolid media. The diameter of the displacement zones increases considerably over time until reaching 38 mm for isolate 7 and 41 mm for *P. aeruginosa* ATCC 27853 (Supplementary material S4).

**Figure 5:**
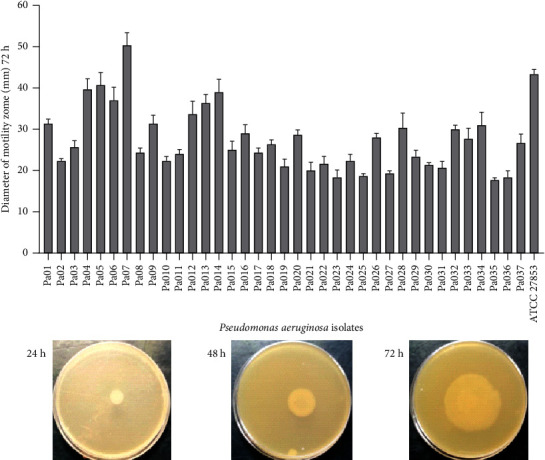
Swimming motility of *P. aeruginosa*. 2.5 *μ*L of bacterial suspension was dropped at the center surface of the agar plate and incubated for 24 h, 48 h, and 72 h at 37°C. In this figure are photographs of representative swimming motility at different time intervals. The diameter of the swimming motility zone increased with incubation time.

The swimming activity of the isolates is shown in Figure 5. All isolates also exhibited the ability to move in fluid or low-viscosity media. The diameter of the halos increases considerably with time and reaches up to 50 mm for isolate 7 (Figure 5) (Supplementary material S5).

### 3.6. Correlation among the Biofilm Formation, Pyocyanin Production, and Antibiotic Resistance

The correlation between the antibiotic sensitivity of *P. aeruginosa* isolates and the concentration of pyocyanin produced is shown in Table 1. The concentration of pyocyanin increased with bacterial resistance. Pearson's coefficient from a correlation between antibiotic resistance and the concentration of pyocyanin production demonstrated a strong correlation for amikacin (*r* = 0.791 and *P* < 0.001), neomycin (*r* = 0.694 and *P* < 0.001), ceftazidime (*r* = 0.846 and *P* < 0.001), and norfloxacin (*r* = 0.758 and *P* < 0.001). No correlation was observed with paromomycin and streptomycin.  Table 2 shows the correlation between antibiotic resistance and biofilm formation.

**Figure 6:**
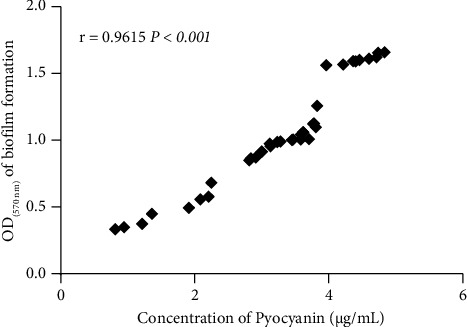
Correlation between biofilm formation and pyocyanin production.

A significant correlation was observed between resistance to amikacin (*r* = 0.77 and *P*=0.001), neomycin (*r* = 0.69 and *P*=0.008), ceftazidime (*r* = 0.74 and *P*=0.001), and norfloxacin (*r* = 0.54 and *P*=0.001) and biofilm formation. The *P* value is less than 0.001. However, no correlation was observed between paromomycin and streptomycin. The biofilm formation and pyocyanin production showed a statistically significant correlation (*r* = 0.96 and *P* < 0.001) (Figure 6).

## 4. Discussion

*P. aeruginosa* causes severe infections, particularly in healthcare settings. It has an outstanding capacity for being selected and for spreading antimicrobial resistance *in vivo* [28]. Our findings confirm that *P. aeruginosa* is present in chronic wounds. The prevalence of *P. aeruginosa* in the wounds of patients at the Dschang District Hospital is 26.1%. *P. aeruginosa* is one of the most common pathogens in chronic wounds, and its ability to form resistant biofilms and several virulence factors that allow the establishment in host tissue, including pyocyanin has been documented [29]. This result corroborates the work done by Raizman et al., who reported the presence of *P. aeruginosa* in an infected wound [4]. However, the prevalence found during this work is lower than in the previous work done by De Oliveira et al. and Raizman et al., who found a prevalence of 72% and 92.9%, respectively, of *P. aeruginosa* in chronic wounds [4, 5]. This difference in prevalence would be due to some factors, such as the sample size, geographical diversity, patient demographical factors, or access and exposure to antimicrobial agents.

Although risk factors for infections with resistant *P. aeruginosa* strains include prior use of broad-spectrum antimicrobials, *Pseudomonas aeruginosa* strains are also known to utilize their high levels of intrinsic and acquired resistance mechanisms to counter most antibiotics. In this study, the antibiotic resistance profile of *P. aeruginosa* isolates was investigated towards six commonly used antibiotics indicated in the treatment of *Pseudomonas* infections. Paromomycin was included since a high resistance of *Pseudomonas aeruginosa* to paromomycin has been previously reported [30]. Given the increasing prevalence of multidrug-resistant *P. aeruginosa* and the paucity of useful antipseudomonal agents, the use of aminoglycosides has become increasingly important in managing *P. aeruginosa* infections. Therefore, in addition to paromomycin, we also included streptomycin as well as amikacin and neomycin. The results of this study showed that 65.8% of the isolates were resistant to at least one agent in three or more antimicrobial categories and, therefore, categorized as MDR. This percentage is lower than that obtained by Moazami Goudarzi and Eftekhar, who showed that 97.4% of *P. aeruginosa* on wounds were MDR [31]. However, De Francesco et al. and Moazami Goudarzi and Eftekhar reported a different prevalence of MDR of *P. aeruginosa* (from 20% to 100%) [31, 32]. The observed discrepancy could be because *P. aeruginosa* infections are complex in pathogenesis and are dependent on various virulence factors such as secretory factors, elastase, phospholipase C, alkaline protease, pyocyanin, hydrogen cyanide, pyoverdine, and rhamnolipids [33]. *P. aeruginosa* also shows an outstanding ability to develop further antimicrobial resistance to all available antibiotics via the acquisition of chromosomal mutations [28]. Bacterial motility plays a key role in the colonization of surfaces by bacteria and the subsequent formation of resistant communities of bacteria called biofilms.

In the present study, 100% of *P. aeruginosa* isolates from infected wounds were biofilm producers, of which 50%, 37%, and 13% were strong, moderate, and weak biofilm producers, respectively. These data support the roles of biofilm wound healing since bacterial biofilm has been recognized as a major factor in delayed wound healing and high levels of biofilm production have been repeatedly described in multidrug-resistant organisms [34]. These results are similar to those of Jabalameli et al., who showed that 96% of *P. aeruginosa* isolated from wounds were biofilm formers of which 49.9% were strong biofilm producers, 26% were moderate biofilm producers, and 22.9% were low biofilm producers [35]. This ability to form a biofilm would be due to a variety of components playing a role in the attachment of *P. aeruginosa* to surfaces. These components include flagella, type IV pili, Cup fimbriae, extracellular DNA (eDNA), and Psl polysaccharides [36]. This ability to form the biofilm would also be due to the release of compounds such as iron siderophores, biosurfactants, and EPS into their surrounding environment.

In this work, all isolates of *P. aeruginosa* were pyocyanin producers, which corroborate the report of Al-Qaysi et al. [23]. Our finding is of great importance for the studied population since pyocyanin is a major virulence factor contributing to *P. aeruginosa* pathogenesis. In other studies, more than 90% of clinical *P. aeruginosa* isolates produce pyocyanin [37]. The correlation observed between the pyocyanin produced and the resistance of *P. aeruginosa* to antibiotics could be due to the important role of pyocyanin in resistance to antibiotics. This result is in agreement with those obtained by Khadim and Marjani, who suggested that pyocyanin itself may play a direct role in the observed link between antimicrobial susceptibilities and the phenotype [38]. Furthermore, Sweedan suggested that pyocyanin production was relatively affected by antibiotic challenges [39]. In addition to the virulence factors produced by *P. aeruginosa*, it is also characterized by its ability to form biofilms.

All *P. aeruginosa* isolates exhibited the ability to move through semisolid (swarming) and low-viscosity (swimming) media. This displacement is due to *P. aeruginosa* flagella which allow colonization of surrounding environments. This work is in agreement with that carried out by Otton et al. [40]. This finding is supported by a report suggesting that in *Pseudomonas aeruginosa*, flagellar stators play several roles in the formation of biofilms and the control of swarming motility [41]. These data were later emphasized by those who suggested a possible structural role for flagella in a biofilm, conceivably as a scaffold [42].

A correlation between pyocyanin production and biofilm formation was observed. This correlation corroborates the study performed by Al-Qaysi et al., who obtained the correlation between pyocyanin production and biofilm formation of *P. aeruginosa* isolates from patients in Iraq [23]. The release of pyocyanin in *P. aeruginosa* clinical strains induces a significant increase in eDNA as a biofilm promoter, which improves biofilm formation [43]. Furthermore, *P. aeruginosa* cells in the presence of eDNA have favorable acid-base interactions, which aid in biofilm production [44]. Moreover, pyocyanin influences essential physicochemical interactions that drive bacterial cell‐to‐cell interactions. Pyocyanin is essential in the ability to foster aggregation. Pyocyanin is characterized by dysregulation of the QS system, leading to increased production of exoproducts, which are essential for the biofilm formation in *P. aeruginosa* [45].

This study sheds light on the biofilm-forming capabilities and pyocyanin production levels of *P. aeruginosa* isolates, a bacterium notorious for its association with chronic wound infections. The results offer valuable insights into the virulence factors associated with *Pseudomonas aeruginosa* infections and contribute substantially to our knowledge of the mechanisms employed by *P. aeruginosa* in evading host defenses, providing a foundation for the development of targeted therapeutic interventions, improving the efficacy of treatments for patients in Dschang Hospital, and potentially influencing wound care practices worldwide. Furthermore, the assessment of antibiotic resistance profiles in these isolates is of critical importance in the era of escalating antibiotic resistance. By determining the resistance patterns of *P. aeruginosa* in the specific context of wounds at Dschang Hospital, the study provides essential information for local healthcare practitioners. This insight allows for more informed antibiotic prescribing practices, promoting effective treatment strategies tailored to the unique resistance profiles observed in the region.

Overall, finding from this study not only contribute significantly to the understanding of *P. aeruginosa* infections in the local context of Dschang Hospital but also offer insights that have broader implications for global infectious disease management. The study's outcomes pave the way for more targeted and effective treatment approaches, reinforcing the importance of locally driven research in shaping the future of healthcare practices worldwide.

## 5. Conclusion

Findings from this study suggest that infected wounds could act as a reservoir for MDR and virulent *P. aeruginosa*. The presence of strong biofilm producers of *P. aeruginosa* in infected wounds is a serious public health concern. Therefore, surveillance programs to monitor and control MDR *P. aeruginosa* in these patients are required to improve patients' quality of life. In addition, biofilm formation and pyocyanin production could also be regarded as potential targets for the development of a new therapeutic agent against *P. aeruginosa* infections. Further studies are needed to investigate the presence of antibiotic resistance and biofilm formation genes as well as the genetic diversity of the isolates.

## Figures and Tables

**Table 1:** Correlation between antibiotic resistance and pyocyanin production.

Antibiotics	Concentration of pyocyanin production (*μ*g/mL)				
	Sensitive	Intermediate	Resistant	*r*	*P* value
Amikacin	2.2 ± 0.8	3.5 ± 0.2	4.2 ± 0.6	0.79	<0.001
Paromomycin	0.0 ± 0.0	0.0 ± 0.0	3.3 ± 1.1	—	—
Streptomycin	0.0 ± 0.0	0.0 ± 0.0	3.3 ± 1.1	—	—
Neomycin	1.1 ± 0.2	0.0 ± 0.0	3.6 ± 0.8	0.69	<0.001
Ceftazidime	1.6 ± 0.6	2.8 ± 0.0	3.8 ± 0.6	0.84	<0.001
Norfloxacin	0.0 ± 0.0	2.0 ± 0.8	3.8 ± 0.7	0.75	<0.001

r: Pearson's correlation coefficient; —: not determined.

**Table 2:** Correlation between antibiotic resistance and biofilm formation.

Antibiotics	OD_(570nm)_ of biofilm formation				
	Sensitive	Intermediate	Resistant	r	*p* value
Amikacin	0.7 ± 0.2	1.0 ± 0.0	1.4 ± 0.3	0.77	<0.001
Paromomycin	0.0 ± 0.0	0.0 ± 0.0	1.0 ± 0.4	—	—
Streptomycin	0.0 ± 0.0	0.0 ± 0.0	1.0 ± 0.4	—	—
Neomycin	0.4 ± 0.0	0.0 ± 0.0	1.1 ± 0.3	0.69	0.008
Ceftazidime	0.5 ± 0.1	0.9 ± 0.0	1.2 ± 0.3	0.74	<0.001
Norfloxacin	0.0 ± 0.0	0.6 ± 0.2	1.2 ± 0.3	0.54	<0.001

r: Pearson's correlation coefficient; —: not determined.

## Data Availability

The datasets generated and/or analyzed during the current study are available from the corresponding author upon reasonable request.

## Supplementary Materials

Supplementary Materials Supplementary materials S1: *Pseudomonas aeruginosa *cell-bound safranin dye. The highest biofilm biomass of *Pseudomonas aeruginosa* was formed after 48 h incubation on microtiter plates and stained with safranin for 15 minutes. Supplementary materials S2: formation of biofilm by *Pseudomonas aeruginosa* at 24 h and 72 h. Supplementary materials S3: production of pyocyanin by *Pseudomonas aeruginosa* at 24 h and 48 h. Supplementary materials S4: swarming mobility of *Pseudomonas aeruginosa* at 24 h and 48 h. Supplementary materials S5: swimming mobility of *Pseudomonas aeruginosa* at 24 h and 48 h.

## References

[1] Heshmatipour Z., Arabameri N., Eftekhar Ardebili S., Jafari Bidhendi Z. (2021). The role of gene mutations (Gyra, parc) in resistance to ciprofloxacin in clinical isolates of *Pseudomonas aeruginosa*. *Iranian Journal of Pathology*.

[2] Moradali M. F., Ghods S., Rehm B. H. A. (2017). *Pseudomonas aeruginosa* lifestyle: a paradigm for adaptation, survival, and persistence. *Frontiers in Cellular and Infection Microbiology*.

[3] Morehead M. S., Scarbrough C. (2018). Emergence of global antibiotic resistance. *Primary Care: Clinics in Office Practice*.

[4] De Oliveira F. P., Pires B. M. F. B., de Cássia Ferreira de Almeida Silva K. (2017). Prevalence, antimicrobial susceptibility, and clonal diversity of *Pseudomonas aeruginosa* in chronic wounds. *The Journal of Wound, Ostomy and Continence Nursing*.

[5] Agnihotri N., Gupta V., Joshi R. M. (2004). Aerobic bacterial isolates from burn wound infections and their antibiograms- a five-year study. *Burns*.

[6] Bessa L. J., Fazii P., Di Giulio M., Cellini L. (2015). Bacterial isolates from infected wounds and their antibiotic susceptibility pattern: some remarks about wound infection. *International Wound Journal*.

[7] Who W. H. O. (2017). Prioritization of pathogens to guide discovery, research and development of new antibiotics for drug-resistant bacterial infections, including tuberculosis. *WHO Report*.

[8] Al-Orphaly M., Hadi H. A., Eltayeb F. K. (2021). Epidemiology of multidrug-resistant *Pseudomonas aeruginosa* in the middle east and North Africa region. *mSphere*.

[9] Li Y., Yang Y., Feng Y., Pu J., Hou L. A. (2021). Combined effects of Pseudomonas quinolone signal-based quorum quenching and graphene oxide on the mitigation of biofouling and improvement of the application potential for the thin-film composite membrane. *Science of the Total Environment*.

[10] Jayaseelan S., Ramaswamy D., Dharmaraj S. (2014). Pyocyanin: production, applications, challenges and new insights. *World Journal of Microbiology and Biotechnology*.

[11] Rashid M. I., Andleeb S., Ali A. (2020). Evaluation of pyocyanin induced systemic pathogenicity of *pseudomonas aeruginosa*. *Pakistan journal of pharmaceutical sciences*.

[12] Abdelaziz A. A., Kamer A. M. A., Al-Monofy K. B., Al-Madboly L. A. (2023). *Pseudomonas aeruginosa*’s greenish-blue pigment pyocyanin: its production and biological activities. *Microbial Cell Factories*.

[13] O’May C., Tufenkji N. (2011). The swarming motility of *Pseudomonas aeruginosa* is blocked by cranberry proanthocyanidins and other tannin-containing materials. *Applied and Environmental Microbiology*.

[14] Ha D. G., Kuchma S. L., O’Toole G. A. (2014). Plate-based assay for swarming motility in *Pseudomonas aeruginosa*. *Methods in Molecular Biology*.

[15] Rajabi H., Salimizand H., Khodabandehloo M., Fayyazi A., Ramazanzadeh R. (2022). Prevalence of algD, pslD, pelF, Ppgl, and PAPI-1 genes involved in biofilm formation in clinical *Pseudomonas aeruginosa* strains. *BioMed Research International*.

[16] Bisso Ndezo B., Tokam Kuaté C. R., Dzoyem J. P. (2021). Synergistic antibiofilm efficacy of thymol and piperine in combination with three aminoglycoside antibiotics against *Klebsiella pneumoniae* biofilms. *The Canadian Journal of Infectious Diseases & Medical Microbiology*.

[17] Bisso B. N., Makuété A. L., Tsopmene J. U., Dzoyem J. P. (2023). Biofilm formation and phospholipase and proteinase production in *Cryptococcus neoformans* clinical isolates and susceptibility towards some bioactive natural products. *The Scientific World Journal*.

[18] Kunwar A., Shrestha P., Shrestha S., Thapa S., Shrestha S., Amatya N. M. (2021). Detection of biofilm formation among *Pseudomonas aeruginosa* isolated from burn patients. *Burns Open*.

[19] Römling U., Balsalobre C. (2012). Biofilm infections, their resilience to therapy and innovative treatment strategies. *Journal of Internal Medicine*.

[20] Tokam Kuaté C. R., Bisso Ndezo B., Dzoyem J. P. (2021). Synergistic antibiofilm effect of thymol and piperine in combination with aminoglycosides antibiotics against four *Salmonella enterica* serovars. *Evidence-based Complementary and Alternative Medicine*.

[21] Ndezo Bisso B., Tokam Kuaté C. R., Boulens N., Allémann E., Delie F., Dzoyem J. P. (2022). Antibiofilm synergistic activity of streptomycin in combination with thymol-loaded poly (lactic-co-glycolic acid) nanoparticles against *Klebsiella pneumoniae* isolates. *Evidence-based Complementary and Alternative Medicine*.

[22] Wu Y. K., Cheng N. C., Cheng C. M. (2019). Biofilms in chronic wounds: pathogenesis and diagnosis. *Trends in Biotechnology*.

[23] Al-Qaysi A. M. K., Al-Ouqaili M. T. S., Al-Meani S. A. L. (2020). Effect of pyocyanin as secondary metabolite on pseudomonal biofilm and in increasing the resistance degree to antipseudomonal agents. *Drug Invention Today*.

[24] Eucast (2020). Routine and extended internal quality control for mic determination and disk diffusion as recommended by eucast. *European Committee on Antimicrobial Susceptibility Testing*.

[25] Horcajada J. P., Montero M., Oliver A. (2019). Epidemiology and treatment of multidrug-resistant and extensively drug-resistant *Pseudomonas aeruginosa* infections. *Clinical Microbiology Reviews*.

[26] Kırmusaoğlu S., Kaşıkçı H. (2020). Identification of ica-dependent biofilm production by *staphylococcus aureus* clinical isolates and antibiofilm effects of ascorbic acid against biofilm production. *Journal of Clinical Pathology*.

[27] Alayande A. B., Aung M. M., Kim I. S. (2018). Correlation between quorum sensing signal molecules and *pseudomonas aeruginosa*’s biofilm development and virulency. *Current Microbiology*.

[28] Breidenstein E. B. M., de la Fuente-Núñez C., Hancock R. E. W. (2011). *Pseudomonas aeruginosa*: all roads lead to resistance. *Trends in Microbiology*.

[29] Muddassir M., Munir S., Raza A. (2020). A Study of isolation and identification of multidrug resistant *Pseudomonas aeruginosa* from wound specimen. *Journal of Pharmaceutical Research International*.

[30] Daxboeck F., Rabitsch W., Stadler M., Assadian O., Leitgeb J. (2013). High resistance of *pseudomonas aeruginosa* to paromomycin, an agent used for selective bowel decontamination (SBD). *GMS hygiene and infection control*.

[31] Moazami Goudarzi S., Eftekhar F. (2015). Multidrug resistance and integron carriage in clinical isolates of *pseudomonas aeruginosa* in tehran, Iran. *Turkish Journal of Medical Sciences*.

[32] De Francesco M. A., Ravizzola G., Peroni L., Bonfanti C., Manca N. (2013). Prevalence of multidrug-resistant *acinetobacter baumannii* and *pseudomonas aeruginosa* in an Italian hospital. *Journal of Infection and Public Health*.

[33] van ‘t Wout E. F. A., van Schadewijk A., van Boxtel R. (2015). Virulence factors of *pseudomonas aeruginosa* induce both the unfolded protein and integrated stress responses in airway epithelial cells. *PLoS Pathogens*.

[34] Di Domenico E. G., Farulla I., Prignano G. (2017). Biofilm is a major virulence determinant in bacterial colonization of chronic skin ulcers independently from the multidrug resistant phenotype. *International Journal of Molecular Sciences*.

[35] Jabalameli F., Mirsalehian A., Khoramian B. (2012). Evaluation of biofilm production and characterization of genes encoding type III secretion system among *pseudomonas aeruginosa* isolated from burn patients. *Burns*.

[36] Ma Q., Wood T. K. (2009). OmpA influences *escherichia coli* biofilm formation by repressing cellulose production through the CpxRA two-component system. *Environmental Microbiology*.

[37] Zhou H., Yang Y., Shang W. (2022). Pyocyanin biosynthesis protects *pseudomonas aeruginosa* from nonthermal plasma inactivation. *Microbial Biotechnology*.

[38] Khadim M. M., Marjani M. F. A. L. (2019). Pyocyanin and biofilm formation in *pseudomonas aeruginosa* isolated from burn infections in Baghdad, Iraq. *Jordan Journal of Biological Sciences*.

[39] G Sweedan E. (2010). Study the effect of antibiotics on pyocyanin production from *pseudomonas aeruginosa* and pyocyanin as antibiotic against different pathogenic bacteria. *Journal of University of Anbar for Pure Science*.

[40] Otton L. M., da Silva Campos M., Meneghetti K. L., Corção G. (2017). Influence of twitching and swarming motilities on biofilm formation in pseudomonas strains. *Archives of Microbiology*.

[41] Toutain C. M., Caizza N. C., Zegans M. E., O’Toole G. A. (2007). Roles for flagellar stators in biofilm formation by *pseudomonas aeruginosa*. *Research in Microbiology*.

[42] Ozer E., Yaniv K., Chetrit E. (2021). An inside look at a biofilm: *pseudomonas aeruginosa* flagella biotracking. *Science Advances*.

[43] Das T., Ibugo A. I., Klare W., Manefield M. (2016). Role of pyocyanin and extracellular dna in facilitating *Pseudomonas aeruginosa* biofilm formation. *Microbial Biofilms- Importance and Applications*.

[44] Das A., Das M. C., Sandhu P. (2017). Antibiofilm activity of: parkia javanica against *pseudomonas aeruginosa*: a study with fruit extract. *RSC Advances*.

[45] Das M. C., Sandhu P., Gupta P. (2016). Attenuation of *pseudomonas aeruginosa* biofilm formation by vitexin: a combinatorial study with azithromycin and gentamicin. *Scientific Reports*.

