# Supplementary material for: Biofilm Formation, Pyocyanin Production, and Antibiotic Resistance Profile of Pseudomonas aeruginosa Isolates from Wounds
Source: Int J Microbiol. 2024 Feb 20;2024:1207536. doi: 10.1155/2024/1207536 (PMC10898945; doi:10.1155/2024/1207536)
Supplement: Supplementary Materials — S1: Pseudomonas aeruginosa cell-bound safranin dye. The highest biofilm biomass of Pseudomonas aeruginosa was formed after 48 h incubation on microtiter plates and stained with safranin for 15 minutes. Supplementary materials S2: formation of biofilm by Pseudomonas aeruginosa at 24 h and 72 h. Supplementary materials S3: production of pyocyanin by Pseudomonas aeruginosa at 24 h and 48 h. Supplementary materials S4: swarming mobility of Pseudomonas aeruginosa at 24 h and 48 h. Supplementary materials S5: swimming mobility of Pseudomonas aeruginosa at 24 h and 48 h. [file 1207536.f1.docx]

**International Journal of Microbiology**

**Biofilm Formation, Pyocyanin Production, and Antibiotic Resistance Profile of *Pseudomonas aeruginosa* Isolates from Wounds.**

Larissa Yetendje Chimi^1^, Borel Ndezo Bisso^1^, Guy Sedar Singor Njateng^1^, Jean Paul Dzoyem^1*^

^1^Department of Biochemistry, Faculty of Science, University of Dschang, P.O. Box: 67 Dschang, Cameroon.

*Correspondence should be addressed to Jean Paul Dzoyem; Tel: +237 699245686. E.mail: [jpdzoyem@yahoo.fr](mailto:jpdzoyem@yahoo.fr) or [jean.dzoyem@univ-dschang.org](mailto:jean.dzoyem@univ-dschang.org)

**Supplementary material**

**S1**: *Pseudomonas aeruginosa* cell-bound safranin dye. The highest biofilm biomass of *Pseudomonas aeruginosa* was formed after 48h incubation on microtiter plates and stained with safranin for 15 min.

**S2:** Formation of biofilm by *Pseudomonas aeruginosa* at 24h and 72h

**S3:** Production of pyocyanin by *Pseudomonas aeruginosa* at 24h and 48h

**S4:** Swarming mobility of *Pseudomonas aeruginosa* at 24h and 48h

**S5:** Swimming mobility of *Pseudomonas aeruginosa* at 24h and 48h

***S1****: Pseudomonas aeruginosa* cell-bound safranin dye. The highest biofilm biomass of *Pseudomonas aeruginosa* was formed after 48h incubation on microtiter plates and stained with safranin for 15 min.

**S2:** Formation of biofilm by *Pseudomonas aeruginosa* at 24h and 72h

**S3:** Production of pyocyanin by *Pseudomonas aeruginosa* at 24h and 48h

**S4:** Swarming mobility of *Pseudomonas aeruginosa* at 24h and 48h

**S5:** Swimming mobility of *Pseudomonas aeruginosa* at 24h and 48h
